# Editorial: Advancements in meningioma management: from imaging techniques to personalized medicine approaches

**DOI:** 10.3389/fneur.2026.1784512

**Published:** 2026-02-23

**Authors:** Terry Lichtor

**Affiliations:** Department of Neurosurgery, Rush University Medical Center, Chicago, IL, United States

**Keywords:** brain tumors, fMRI, management, meningiomas, SYHA1813, tranexamic acid, visual outcome

Meningiomas can lead to neurologic deficits including progressive visual loss due to the nerve's complex intra-orbital, intracanalicular, and intracranial anatomy. This Research Topic explores the impact of surgery and treatment strategies in patients with meningiomas. Intracranial meningiomas account for a significant proportion of intracranial neoplasms. Although these tumors have a lower malignancy rate than most intracranial neoplasms, they can exhibit aggressive behavior with a likelihood of recurrence. This Research Topic examines issues regarding patient demographics, innovative diagnostic techniques, novel prognostic markers, and effective treatment strategies.

Filimonova et al., in a research report, *Reorganization of brain networks in olfactory groove meningioma patients: a pilot resting-state fMRI study*.

Olfactory groove meningiomas are frequently associated with neuropsychological and behavioral impairments. The etiology of these changes is unclear. In this study it was found that there are significant alterations in the frontal-parietal networks as detected by resting-state fMRI data processing compared to controls that appear to be associated with clinical variables and lesion characteristics. These findings were correlated with edema in the region of the tumor along with the neurologic exam findings.

Krahulik et al., in a research report, *Visual outcomes in patients with meningiomas compressing optic nerve*.

In some meningiomas where visual impairment is an issue, it has been found that surgery to decompress the optic nerve compressed by the meningioma irrespective of the tumor size results in improved long-term recovery of vision. The findings in this study also suggest that the length of time that the patient experiences visual symptoms before surgery is significant in the outcome, although other factors such as tumor size are not directly associated with eventual gain of visual acuity.

Kong et al., in a research report, *Risk factors for postoperative thrombotic complications after meningioma resection: a retrospective single-center study in China*.

Post-operative venous thromboembolism and pulmonary embolism are major causes of morbidity and mortality following resection of meningiomas. The exact pathophysiology for the increased risk of developing deep venous thrombosis following resection of these tumors is unclear. The development of post-op thromboembolic events is associated with the age of the patient along with other comorbidities which need to be considered prior to surgery.

Lan et al., in a research report, *A novel compound, SYHA1813, inhibits malignant meningioma growth directly by boosting p53 pathway activation and impairing DNA repair*.

Novel antitumor agents are being developed for treatment of meningiomas. Results have shown that the unique agent SYHA1813 can directly attack and inhibit the growth of meningioma tumor cells both *in vitro* and *in vivo*. This is a promising therapeutic agent that boosts p53 pathway activation and impairs DNA repair. This agent has been used in a clinical trial of patients with recurrent meningioma with evidence of anti-tumor activity.

Vychopen et al., in a systemic review, *Intraoperative tranexamic acid administration in cranial meningioma surgery: a meta-analysis of prospective randomized, double-blinded, and placebo-controlled trials*.

Cranial meningioma surgery also often involves significant blood loss. A single intraoperative dose of tranexamic acid, which is an antifibrinolytic agent, has been shown to reduce blood loss and blood transfusions along with shortening surgery time.

Tleubergenov et al., case report, *Simultaneous surgical management of a giant tuberculum sellae meningioma and pregnancy-related complications: a case report and literature review*.

Meningiomas pose an unusual challenge during pregnancy. Their growth can increase due to hormonal and hemodynamic changes which require balance for maternal and fetal risks. On occasion neurosurgical and obstetric procedures are done simultaneously when clinically indicated for optimal outcome. A carefully planned approach is necessary for these patients with meningiomas who are pregnant to ensure the optimal outcome for both the fetus and mother.

Pu et al., case report, *Surgical treatment of primary intracranial and extracranial communication leiomyosarcoma: a case report*.

Primary intracranial-extracranial communicating leiomyosarcomas, capable of invading both intracranial and extracranial regions and involving complex anatomical structures, are exceedingly rare neoplasms with surgery as the optimum treatment. The decision to remove the lesion should involve issues including tumor location and evidence of distant metastases.

Zhenwei et al., case report, *Primary subcutaneous Rosai-Dorfman-Destombes of the scalp with intra-cranial involvement: diagnosis and treatment of a rare case with literature review*.

There are other disorders such as Rosai-Dorfman-Destombes Disease (RDD) which is an uncommon proliferative disorder of histiocytes with features and clinical findings like those of intracranial meningiomas. This disorder is associated with primary scalp and intracranial involvement, and both CT and MRI scans in these lesions often show scalp and intracranial extension. This disorder is a rare problem that would require a collaborative effort with a radiologist and pathologist for appropriate evaluation and surgical planning. The imaging should be carefully reviewed for evidence of involvement of the cranial bones and extracranial involvement. Extensive surgery can often lead to total removal of this tumor.

