# Correction: Editorial: Advancements in meningioma management: from imaging techniques to personalized medicine approaches

**DOI:** 10.3389/fneur.2026.1923253

**Published:** 2026-08-24

**Authors:** Terry Lichtor

**Affiliations:** Department of Neurosurgery, Rush University Medical Center, Chicago, IL, United States

**Keywords:** brain tumors, fMRI, management, meningiomas, SYHA1813, tranexamic acid, visual outcome

In the published article, there was an error. A paragraph summarizing an unrelated research report was included in the Editorial.

The following text has been removed from the Editorial:

“*Du, in a research report, Leveraging named entity recognition for enhanced meningioma management: integrating imaging and personalized medicine data*.

*Advanced computational techniques have been shown to be important in the development of treatment strategies. As diagnostic and treatment paradigms evolve, there is a growing emphasis on leveraging high-throughput data and artificial intelligence to inform clinical decision-making. Traditional Named Entity Recognition (NER) methods, often reliant on rule-based systems or conventional machine learning algorithms, struggle with the complexity and variability inherent in medical texts. Recent advancements in deep learning and transformer-based language models offer promising alternatives by enabling context-aware recognition and improved generalization across varied datasets. Integrating models into biomedical pipelines could significantly enhance the extraction of meaningful information, ultimately facilitating more precise and individualized approaches to meningioma care*.”

The original version of this article has been updated.

